# Comparing the fatigability of paraspinal muscles between sides and curve levels while performing a modified side plank in patients with adolescent idiopathic scoliosis

**DOI:** 10.1186/1748-7161-9-S1-O11

**Published:** 2014-12-04

**Authors:** Alan Richter, Eric C Parent, Gregory Kawchuk, Marc Moreau

**Affiliations:** 1 University of Alberta, Edmonton, Canada

## Background

While many studies have been performed on the effects of exercise on Adolescent Idiopathic Scoliosis (AIS) little research has been done into the underlying muscle physiology. Studies have suggested a fibre type imbalance, however, the implications of this difference as reflected through fatiguability have not been studied. The slope of the median frequency of the surface electromyographic (EMG) signal is a useful tool in determining the fatigability of muscles.

## Aim

To compare the fatigability of paraspinal muscles between levels and sides in patients with AIS.

## Design

Cross-sectional study

## Methods

Subjects with AIS were recruited from our specialized scoliosis clinic. Subjects performed 3 'modified side planks' on both left and right sides. Bipolar sEMG electrodes were placed on either side of the spine at the upper end vertebrae(UEV), apex, and lower end vertebrae(LEV). Raw EMG muscle activity was recorded. The slope of the median frequency of the EMG power spectrum was extracted using Matlab. A repeated measures side-by-level ANOVA was performed to detect differences in the average of the closest 2 out 3 fatigue trials between sides and levels. A paired t-test was performed to determine if there were differences in trial duration between left and right planks

## Results

Thirteen subjects were recruited (10 females) with a mean age of 13.6±1.6 years and a mean BMI of 19.76±3.8kg/m^2^. Mean Cobb angle was 25±10.3 degrees. No significant interaction or main effects were found in fatigue measurements between sides and levels. Mean slope of median frequency over sides and levels was 0.023±0.103 for left plank and 0.108±0.21 for right plank. There were no significant differences between left (55.46sec±19.39) and right (58.31sec±19.61) plank mean durations.

## Conclusions

The side plank did not create enough fatigue in paraspinal muscles when using sEMG as to measure the slope of the median frequency to detect differences between levels and sides. Future work will address the study objectives using the Sorensen test to generate more paraspinal fatigue.
